# Addressing tensions when popular media and evidence-based care collide

**DOI:** 10.1186/1472-6947-13-S3-S3

**Published:** 2013-12-06

**Authors:** Gary Schwitzer

**Affiliations:** 1 HealthNewsReview.org, Saint Paul, MN 55116, USA; 2 University of Minnesota School of Public Health, Minneapolis, Minnesota, 55455, USA

## Abstract

**Background:**

Health care news stories have the potential to inform and educate news consumers and health-care consumers about the tradeoffs involved in health-care decisions about treatments, tests, products, and procedures. These stories have the potential to influence not only individual decision making but also the broader public dialogue about health-care reform. For the past 7 years, a Web-based project called http://HealthNewsReview.org has evaluated news stories to try to improve health-care journalism and the quality and flow of information to consumers.

**Analysis:**

http://HealthNewsReview.org applies 10 standardized criteria to the review of news stories that include claims about medical interventions. Two or three reviewers evaluate each story. The team has evaluated more than 1,800 stories by more than a dozen leading U.S. news organizations. About 70% have received unsatisfactory scores based on application of these criteria: reporting on costs, quantifying potential benefits, and quantifying potential harms.

**Conclusions:**

Inaccurate, imbalanced, incomplete news stories may drown out more careful scrutiny of the evidence by many influential news organizations. Unquestioned claims and assertions about the benefits of medical interventions are passed on to the American public daily by journalists who are supposed to vet independently any such claims. Communication about these issues is, in itself, a major health-policy issue.

## Background

At a time when many Americans may be hearing about comparative effectiveness research for the first time, many health care news stories by U.S. news media emphasize or exaggerate the potential benefits of interventions while minimizing or ignoring the potential harms. Ironically, as the health-care reform bill in the United States was named the Patient Protection and Affordable Care Act (Public Law 11-148), most of the news stories released by the U.S. news media about medical interventions failed to adequately discuss cost—or affordability—issues.

In an era when many health insurance companies are marketing high-deductible or so-called consumer-driven health-care plans predicated on the assumption that consumers will be more cost-conscious if they receive more information about the cost of care and quality of their providers, much of the news and information they receive every day through mass media messages doesn’t help them define or assess such quality. While evidence-based medicine proponents promote a shared decision-making discussion of the tradeoffs between harms and benefits in screening test decisions, the public dialogue may be overtaken by proscreening advocates who treat screening as a mandate, not a choice.

Deyo and Patrick wrote, “Vested interests, marketing, politics and media hype often have more influence on how new medical advances get used than the best scientific evidence” [1]. The intersection of medicine and the media is often a messy place, and the public may be harmed as a result.

This paper will document recurring problems in media messages about health-care interventions and will offer suggestions for new or expanded efforts to improve the public dialogue about health care.

## Analysis

This paper will review the findings from http://HealthNewsReview.org, a unique project that evaluates and tries to improve the quality of health-care media messages. It will also provide a closer look at some specific problems in health-care journalism, including some that were observed in news coverage of the revised mammography recommendations released by the U.S. Preventive Services Task Force (USPSTF) in 2009.

For 7 years, the http://HealthNewsReview.org project has evaluated news stories about healthcare interventions (treatments, tests, products, procedures) by leading news organizations in the United States. Ten standardized criteria are applied to the review of each story. These criteria represent journalism issues identified either in a *New England Journal of Medicine* paper in 2000 [2] or in the Association of Health Care Journalists “Statement of Principles” [3].

The criteria for analysis include these questions:

**•** Does the story adequately discuss the costs of the intervention?

**•** Does the story adequately quantify the benefits of the treatment/test/product/procedure? (A story that frames benefits only in relative [not absolute] risk reduction terms is likely to get an unsatisfactory score.)

**•** Does the story adequately explain/quantify the harms of the intervention?

**•** Does the story seem to grasp the quality of the evidence?

**•** Does the story commit disease-mongering?

**•** Does the story use independent sources and identify conflicts of interest in sources?

**•** Does the story compare the new approach with existing alternatives?

**•** Does the story establish the availability of the treatment/test/product/procedure?

**•** Does the story establish the true novelty of the approach?

**•** Does the story appear to rely solely or largely on a news release?

Data from the http://HealthNewsReview.org project appear in Table 1.

**Table 1:** Results from review of 1,854 health news stories by HealthNewsReview.org

Criterion	No. of satisfactory scores	No. of unsatisfactory scores	No. of not applicable (N/A) scores	Percentage of satisfactory scores (total minus N/A)
Costs	476	1,087	291	30%
Benefits	623	1,204	27	34%
Harms	626	1,158	70	35%
Evidence	713	1,137	4	39%
Disease-mongering	1,385	397	72	78%
Sources/conflict of interest	1,021	829	4	55%
Alternatives	781	1,021	52	43%
Availability	1,227	450	177	76%
Novelty	1,372	381	101	78%
Rely on press release*	1,329	120	405	92%

* The criterion asking whether the story appeared to rely solely or largely on a news release requires some explanation. Note the high number of not applicable (N/A) scores. In order to make a judgment on this criterion, reviewers must have a copy of a news release. Because a news release is not always available, many stories are graded N/A. The percentage of satisfactory scores may seem high; another perspective is that the fact that 120 supposedly independently reported stories were found to rely solely or largely on a news release is troubling.

Each story is reviewed by two to three reviewers from a team of people trained to evaluate medical evidence [4]. Journalists are notified by email whenever one of their stories is reviewed.

## The daily drumbeat of imbalanced messages

About two out of every three news stories reviewed fail to adequately address costs and fail to adequately quantify harms and benefits. For an American audience that may not even be aware that its nation spends a world-leading 17% of its gross domestic product on health care [5], leaving more than 16% of the population uninsured [6], journalists may not be delivering vital information on important public policy issues. The dissonance between comparative effectiveness research and daily media messages that emphasize or exaggerate the benefits of interventions and minimize or ignore their harms is striking.

Recurring themes in these media misadventures include:

**•** Stories may frame benefits in the most positive light by using relative risk reduction statistics without the corresponding absolute risk reduction numbers.

**•** The tyranny of the anecdote: Stories may use only positive, glowing patient anecdotes but fail to mention trial dropouts, compliance problems, patient dissatisfaction, or the choice to pursue less aggressive options.

**•** Stories may fail to explain the limitations of observational studies and conflate association with causation. Misreporting of observational studies may lead readers to question the credibility of both journalism and science.

**•** Stories may frame surrogate markers or intermediate endpoints as if they were outcomes on which consumers should focus.

**•** Many news organizations seem to believe that anything published in a medical journal is infallible and unquestionably newsworthy. Journalists should become familiar with the work of Stanford University professor John Ioannidis, such as “Why Most Published Research Findings Are False” [7]. Increasingly, many news organizations cover more stories out of journals each week—often as a cost-savings measure because reporters can cover these stories without leaving the newsroom. But journalists who feed off of a steady diet of studies published in journals may not be aware of the publication bias in favor of positive findings in many journals. So that steady diet of journal stories may convey to readers an overly optimistic view of research progress.

**•** About half of all stories reviewed are single-source stories and/or those that fail to disclose conflicts of interest in sources. This approach should not give readers confidence in the balance and integrity of the story.

## An uninformed public dialogue about screening

What was seen in much (not all) news coverage of the November 2009 recommendations of the USPSTF on screening for breast cancer was disturbing because of the imbalanced coverage in many news stories. Many stories demonstrated a lack of knowledge about the USPSTF, a lack of appreciation for the expertise of its members, and failure to grasp the fact that screening tests may carry different harms and benefits for different age groups. The framing of many stories was built on themes of a government task force trying to save money or ration care, of government deciding that “some lives don’t matter,” or of personal anecdotes of women who claimed their lives had been saved by mammograms. In the patient or consumer interviews some stories chose to highlight, people said they were shocked by the recommendations because they “seemed to come out of nowhere” or “out of the blue.” Many stories did little to even hint at the long historical context of the ongoing uncertainty over the benefits and harms of breast cancer screening, especially in younger women. Finally, some stories were framed around messages that said the “shifting recommendations prove that scientists are clueless”; others questioned the expertise of USPSTF members because none had subspecialty training in oncology or radiology.

The important shared decision-making message of the recommendations published by the USPSTF never appeared in many stories, columns or talk shows that followed. The USPSTF wrote: “The decision to start regular, biennial screening mammography before the age of 50 years should be an individual one and take patient context into account, including the patient's values regarding specific benefits and harms.” That message was either missed or ignored in many stories and became a topic of ridicule in other instances—almost as if uncertainty was something to be shunned at all costs.

The editors of the *Annals of Internal Medicine* published an editorial, “When Evidence Collides With Anecdote, Politics, and Emotion: Breast Cancer Screening” [8], in which they referred to a “media cacophony”:

“…[T]he media and politicians presented the breast cancer screening recommendations as a major departure from existing guidelines that heralded an age of rationed care in the United States. Confusion, politics, conflicted experts, anecdote, and emotion ruled front pages, airwaves, the Internet, and dinner-table conversations.…

The initial reaction to the Task Force recommendations might have been less vehement had the potential negative consequences of alternative recommendations also been considered.”

The true potential harms of screening were rarely communicated. Veteran science journalist John Crewdson wrote in *The Atlantic *[9]:

“The current controversy over the task force's report owes much to the media's confusing coverage, some of which has been misinformed, including by TV doctors who ought to know better.

…There are multiple reasons women are ill-informed about breast cancer. The fault lies primarily with their physicians, the cancer establishment, and the news media—especially the news media. Until coverage of breast cancer rises above the level of scary warnings mixed with heartwarming stories of cancer survivors, women are likely to go on being perplexed.”

The challenge for those communicating evidence-based health-care messages is clearly shown in this one episode from our recent history. But it has been repeated with news coverage of evidence-based statements on prostate cancer screening, lung cancer screening, and coronary artery disease screening.

## Public confusion

The Kaiser Family Foundation Health Tracking Poll shows that Americans are confused about much of what they hear and read about health care [10]. In a May 2010 poll, the Kaiser Family Foundation found that 44% of those polled were confused about the health-care reform legislation. Journalists play a role in creating that confusion.

Of Americans polled, 63% got health-care information from cable TV news programs and 55% from broadcast news programs. From the Kaiser Family Foundation report: “Further breaking down those getting health reform information from cable news, 25 percent of Americans indicated their main cable source on this topic was FOX News, 22 percent named CNN and 6 percent MSNBC. In fact, cable news still tops the list of the public’s ‘most important’ sources of news about the new law, with 30 percent saying they rely on that source more than any other.”

Public confusion is fueled, not helped, by journalists who promote unquestioned claims and assertions with a combination of naïveté, anecdotes, emotions, and editorializing.

## Conclusions

A call to action could arise from a recent study published in *Health Affairs*, “Evidence That Consumers Are Skeptical About Evidence-Based Health Care” [11]. Its findings were chilling:

“…[O]ur findings illuminate real and significant challenges to the pursuit of broader acceptance of evidence-based health care among consumers. The beliefs underlying the themes that surfaced in both the qualitative research and the survey—more is better, newer is better, you get what you pay for, guidelines limit my doctor’s ability to provide me with the care I need and deserve—are deeply rooted and widespread. Our findings, although preliminary, have implications for several public and private efforts. These efforts intend to foster—and to some degree depend on—consumer engagement, or at least on the absence of overt consumer resistance.”

Many of the concepts that need to be communicated to the public under the umbrella of evidence-based guideline-setting are counterintuitive. The fact that they are counterintuitive should be addressed early, often, and clearly. Decades of “first impressions” from other sources have molded the thinking of many people. For those who were raised being told they should fight cancer “with a checkup and a check,” it may be very difficult to grasp that what is often framed as “a simple blood (or other screening) test” may actually carry potential harms as well as benefits. Communicators need to learn how to communicate about the tradeoffs of harms and benefits in screening tests and treatment decisions and about shared decision making that allows the individual to address his/her own values in the face of uncertainties.

## Some steps that could be taken

Some excellent journalism training opportunities exist. Others have been shut down or been threatened by lack of funding. The following is an outline of activities that funders could consider.

## • Government-funded efforts

After the November 2009 mammography screening episode, it was clear that many journalists and many citizens have little knowledge about the USPSTF, its mission, or its members. The Agency for Healthcare Research and Quality (AHRQ), a U.S. federal government research agency, could launch an educational outreach effort about the work of the USPSTF. It would seem that the Patient-Centered Outcomes Research Institute—“authorized by [the U.S.] Congress to conduct research to provide information about the best available evidence to help patients and their health care providers make more informed decisions”—would also want to invest in educational efforts for journalists and the general public.

The National Institutes of Health have been very successful in training journalists in the United States how to scrutinize medical evidence in their annual media workshops [12]. (Note: The 2013 Medicine in the Media course was cancelled due to federal budget sequestration.) For 14 years, the Rocky Mountain Workshop on How to Practice Evidence-Based Health Care, with AHRQ support, has often invited journalists to learn about medical evidence and share ideas and challenges with physicians, public health professionals, and policymakers [13].

## • Foundation efforts

The Knight Foundation supports an annual Medical Evidence Boot Camp at the Massachusetts Institute of Technology (MIT). The California Endowment funds training, Web publishing and fellowships at the University of Southern California’s Annenberg School for Communication and Journalism. The investigative journalism of ProPublica’s newsroom (http://www.propublica.org)—much of it on health care issues—is supported by the Sandler Foundation, the Knight Foundation, the MacArthur Foundation, the Pew Charitable Trusts, the Ford Foundation, and the Carnegie Corporation.

Foundations could do more. It seems that for every specialized science/medical/health journalism graduate program that has survived (e.g., University of Georgia, University of North Carolina-Chapel Hill, New York University, Boston University, University of California-Santa Cruz, Columbia University, MIT), others are shut down for apparent economic reasons (the University of Minnesota master’s program in health journalism, the Johns Hopkins University master’s program in science writing, and the Florida Atlantic University science writing program). Foundations could help support such efforts.

http://HealthNewsReview.org was supported by the Informed Medical Decisions Foundation for 8 years, but that funding ends in 2013 with no replacement funding in place at this time. Its German counterpart, Medien-Doktor (http://www.medien-doktor.de/english/) benefits from foundation support. But the pioneering effort of this genre, Media Doctor Australia (http://www.mediadoctor.org.au/), stopped publishing in 2012 because of lack of funding, the same fate that befell a Media Doctor Canada project earlier. Foundations could help these efforts as well.

## • Not-for-profit organization layperson training effort

Another model could be the popular Project LEAD science training course offered by the National Breast Cancer Coalition (NBCC) to its members [14]. Project LEAD courses prepare attendees to understand the basics of breast cancer science, how the biomedical research process works, and how to more accurately understand scientific information in the news.

At an Institute of Medicine workshop, Fran Visco, president of NBCC, criticized the USPSTF’s communication and dissemination strategies outlined in a 2007 report [15]. She said:

“Unfortunately, these strategies focused on dissemination to professional audiences via medical journals and federal agencies, professional societies, and quality improvement organizations via public meetings as the primary audiences. As the recent breast cancer screening example illustrates, strategies for disseminating information to the media, policy makers, and the public are also crucial components of any communication plan.”

## What if? What if not?

If evidence-based medicine advocates can make progress in any of the areas described above, perhaps they will gain a foothold in advancing meaningful health-care reform.

If these advocates fail to make progress, the authors of a recent *Health Affairs* paper provide a somber warning:

“To the extent that consumers perceive that the application of comparative effectiveness research to decision making could limit their choice of providers, inappropriately interfere with physicians’ recommendations for treatment, or appear to "ration" care based on cost, these efforts will encounter consumer resistance and could lead to a broad consumer backlash.…

Effective communication with and support of consumers is essential to improving the quality of health care and containing health care costs. Clearly, consumers will revolt if evidence-based efforts are perceived as rationing or as a way to deny them needed treatment. Policy makers, employers, health plans, providers, and researchers will thus need to translate evidence-based health care into accessible concepts and concrete activities that support and motivate consumers. A necessary condition for effective communication, after all, is to start where your audience is—even if that is not where you hoped or expected it to be” [11].

It is clear that many U.S. citizens are not where evidence-based health-care proponents wish they would be. And it’s also clear that many who balk at these principles gravitate toward the benefits of interventions that have been so successfully drummed into them and are deaf to the potential harms and/or absence of benefit that have been underemphasized or ignored entirely in many forms of communication.

Independent of the delivery of care, communication about these issues to and with the American public—by government agencies, providers, researchers, medical journal editorial boards, advocacy groups, the drug and device industry, and journalists—is, itself, a major health-policy issue.

## Abbreviations used

MIT: Massachusetts Institute of Technology; NBCC: National Breast Cancer Coalition; USPSTF: U.S. Preventive Services Task Force

## Competing interests

None.

## Declarations and disclaimer

The Eisenberg Conference Series 2012, *Supporting Informed Decision Making When Clinical Evidence and Conventional Wisdom Collide*, was conducted in Rockville, Maryland, by the John M. Eisenberg Center for Clinical Decisions and Communications Science, Baylor College of Medicine, Houston, Texas, for the Agency for Healthcare Research and Quality under Contract No. HHSA 290-2008-10015-C. Publication costs for this supplement were funded by this contract. The author of this article is responsible for its content. No statement may be construed as the official position of the Agency for Healthcare Research and Quality and of the U.S. Department of Health and Human Services.

This article has been published as part of *BMC Medical Informatics and Decision Making* Volume 13 Supplement 3, 2013: Articles from the Eisenberg Center Conference Series 2012: Supporting informed decision making when clinical evidence and conventional wisdom collide. The full contents of the supplement are available online at http://www.biomedcentral.com/bmcmedinformdecismak/supplements/13/S3.
